# Pd(II) complexes of acetylcholinesterase reactivator obidoxime

**DOI:** 10.2478/intox-2014-0019

**Published:** 2014-12-30

**Authors:** Ahmed Nedzhib, Silviya Stoykova, Vasil Atanasov, Ivayla Pantcheva, Liudmil Antonov

**Affiliations:** 1 Laboratory of Biocoordination and Bioanalytical Chemistry, Department of Analytical Chemistry, Faculty of Chemistry and Pharmacy, “St. Kl. Ohridski” University of Sofia, Sofia, Bulgaria; 2 Emergency Toxicology Clinic, Military Medical Academy, Sofia, Bulgaria; 3 Institute of Organic Chemistry with Center of Phytochemistry, Bulgarian Academy of Sciences, Sofia, Bulgaria

**Keywords:** obidoxime, Pd(II) complex, paraoxon inhibition, acetylcholinesterase reactivation

## Abstract

The ability of the acetylcholinesterase reactivator obidoxime (H_2_L^2+^) to bind palladium(II) cations was evaluated spectrophotometrically at different reaction conditions (pH, reaction time, metal-to-ligand molar ratio). The results showed that immediately after mixing the reagents, pH 7.4, complex species of composition [PdHL]^3+^ existed predominantly with a value of conditional stability constant lg*β*‘=6.52. The reaction was completed within 24 hours affording the formation of species [Pd_2_L]^4+^ with significantly increased stability (lg*β*‘=9.34). The spectral data suggest that obidoxime coordinates metal(II) ions through the oximate functional groups. The *in vitro* reactivation assay of paraoxon-inhibited rat brain acetylcholinesterase revealed that the new complex species were much less active than the non-coordinated obidoxime. The lack of reactivation ability could be explained by the considerable stability of complexes in solution as well as by the deprotonation of oxime groups essential for recovery of the enzymatic activity.

## Introduction

Acetylcholinesterase (acetylhydrolase, AChE, EC 3.1.1.7) is a serine protease that hydrolyzes the neurotransmitter acetylcholine (ACh). During neurotransmission, ACh is released from the terminal of the nerve cell (synapse) and binds to ACh receptors, relaying the signal from the nerve to the muscle. Inhibition of AChE leads to accumulation of ACh and results in impeded neurotransmission and consequent neuromuscular block.

Organophosphorus compounds (OPC) are a class of irreversible AChE inhibitors forming a covalent bond between the phosphoryl group and the esteratic site of the enzyme. The inhibition of AChE leads to muscular paralysis, convulsions, bronchial constriction, and death by asphyxiation (Marrs, 1993; Bajgar, 2004).

The chemical antidotes used for treatment of OPC intoxications are known as cholinesterase reactivators (ChR). The most effective ChR represent mono- or bis-quaternary pyridinium aldoximes containing substituents at various positions in pyridinium rings and/or different bridge type/length between rings. There are some difficulties in application of ChR due to their fast elimination and to the non-complete recovery of enzymatic activity. Moreover, the antidotal activity of reactivators is different against various OPC and universal antidotes have not yet been developed (Kovarik *et al.*, 2006; Musilek *et al.*, 2007; Petroianu *et al.*, 2007a).

From the chemical point of view, the oxime-containing compounds represent potential ligands able to bind metal ions. Their coordination could be used as a strategy to increase the efficacy of active oxime species in the organism since it is known that inclusion of metal ions into the structure of organic molecules could enhance the biological properties of starting ligands. For example, Cier *et al.* (1969) found that the presence of some divalent metal ions potentiated the activity of the acetylcholinesterase reactivator pralidoxime, possibly by the formation of corresponding complex species. To prove whether metal ions can interfere with antidote therapy in case of OPC intoxications, we initiated a broad study assessing the ability of various pyridinium aldoximes to coordinate metal ions.

**Figure 1:**
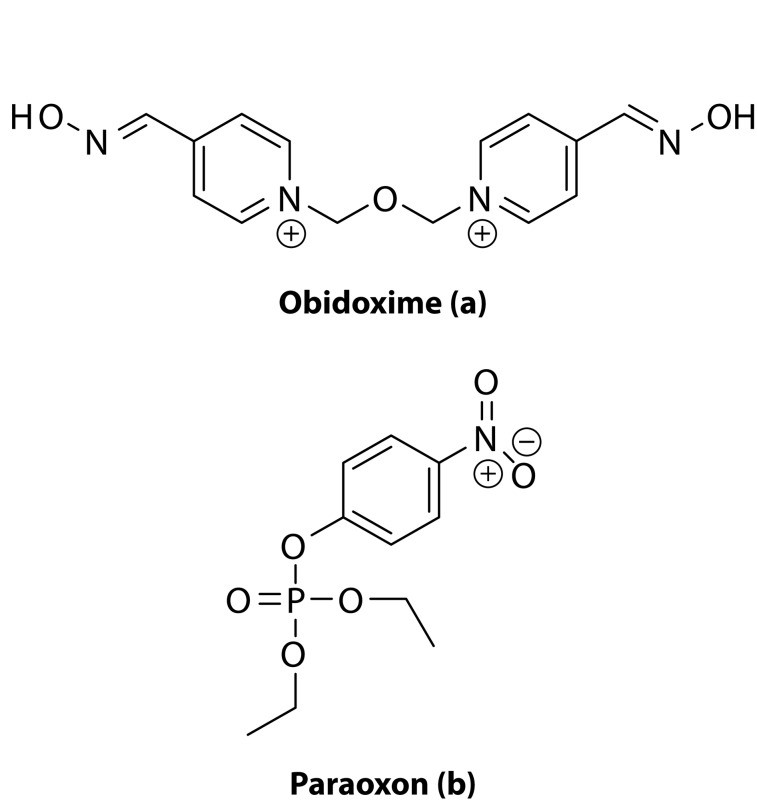
Chemical structures of obidoxime (a) and paraoxon (b).

In the present paper we report the results on the reaction system containing obidoxime (Figure 1a) and palladium(II) cations. The *in vitro* effect which this metal-ligand system renders on reactivation of rat brain acetylcholinesterase inhibited by the organophosphorus insecticide paraoxon (Figure 1b) is described as well.

## Materials and methods

Ammonium tetrachloropalladate(II) ((NH_4_)_2_PdCl_4_), paraoxon (diethyl 4-nitrophenyl phosphate), acetylthiocholine iodide and dithiobisnitrobenzoic acid (DTNB) were obtained from Sigma-Aldrich (Germany). Britton-Robinson buffers (pH 6.3, 7.4, 8.0) (Britton & Robinson, 1931; Coch-Frugoni, 1957) were freshly prepared before experiments by mixing 8×10^–2^ M phosphoric acid, boric acid and acetic acid with an appropriate volume of 4×10^–2^M NaOH. All reagents were of analytical grade; deionized water (18.2 MΩ.cm) was used in experiments.

Obidoxime (1,1’-(oxydimethylene)bis(pyridinium-4-carbaldoxime), H_2_L^2+^) and frozen rat brain (male Wistar rat, 180–220 g) serving as a source of acetylcholinesterase were kindly provided by the Military Toxicology Research Laboratory, Military Medical Academy, Bulgaria.

### Spectrophotometric study on the reaction system palladium(II) – obidoxime

The complexation between Pd(II) ions and obidoxime (H_2_L^2+^) at different reaction conditions (pH, molar ratio of reagents, reaction time) was studied by UV-Vis spectroscopy in the range from 240 to 470 nm. The stock solutions of reagents (4×10^–2^ M) were prepared in Britton-Robinson buffer (pH 6.3, 7.4 or 8.0). Appropriate volumes of these solutions were mixed in the corresponding Britton-Robinson buffer to obtain series of solutions containing metal-to-ligand molar ratio varying from 1:10 to 10:1 in a final volume of 1 mL. The final concentration of obidoxime was kept constant at 4×10^–5^ M. All spectra were recorded against solutions containing corresponding concentrations of palladium(II) salt. The spectral changes were followed up immediately after mixing the reagents, and in case of pH 7.4, up to one week. A Shimadzu UV-1800 spectrophotometer was used in the experiments.

The absorption spectra of the mixture Pd(II) ions – obidoxime (H_2_L^2+^) at different reaction conditions (pH, molar ratio of reagents, reaction time) were processed using the approach for quantitative analysis of undefined mixtures FiNAl (Fishing Net Algorithm), whose mathematical background was described previously (Antonov & Nedeltcheva, 1996; Antonov & Petrov, 2002).

### *In vitro* inhibition of rat brain acetylcholinesterase

Rat brain was weighed and mixed with deionized water to obtain 10% (w/w) brain homogenate using tissue homogenizer and ice bath. The homogenate was centrifuged (5 000 rpm, 10 min) and aliquots from supernatant were collected and stored at 4 °C (up to 4 hours) or at –20 °C (up to 1 month). For inhibition reactions 2% brain homogenate (freshly prepared in Britton-Robinson buffer, pH 7.4) was used.

Series of paraoxon solutions with different concentrations (from 1×10^–4^ M to 1×10^–8^ M final concentration) were prepared in water using stock ethanolic solution (1 mg/mL, 3.6×10^–3^ M). Inhibitor solution (50 µL) was added to the brain homogenate (2%, 450 µL); the reaction mixtures were vortexed and incubated at 25 °C for 30 min. The samples were stored in ice bath until performance of acetylcholinesterase assay (*EA*_*inh*_). A brain homogenate sample containing water instead of inhibitor solution served as control (*K*_*av*_). All samples were prepared in triplicate and measured in duplicate. Results are presented as means and their standard deviations.

The inhibition effect was calculated according to Eq. (1).

1 $$\%\text{Inhibition}=(1-\frac{EA_{\text{inh}}}{K_{\text{av}}}).100$$

The concentration of paraoxon causing 60–70% inhibition of acetylcholinesterase activity was further used in reactivation experiments.

### *In vitro* reactivation of inhibited acetylcholinesterase

Obidoxime solution and a mixture containing Pd(II) ions at molar ratio of Pd(II)-obidoxime = 2:1 were prepared in Britton-Robinson buffer (pH 7.4). Individually these solutions were added (50 µL) to a solution of inhibited rat brain acetylcholinesterase (450 µL), at final concentration of obidoxime 4×10^–5^ M. The reaction mixtures were vortexed and incubated at 25 °C for 30 min. They were stored in ice bath before acetylcholinesterase activity measurements (*EA*_*react*_). The samples containing buffer instead of reactivation solution served as controls (*EA*_*inh*_). The effect of Pd(II) salt (8×10^–5^ M) was also determined. All samples were prepared in triplicate and measured in duplicate. Results are presented as means and their standard deviations.

The reactivation effect was calculated according to Eq. (2).

2 $$\%\text{Reactivation}=(1-\frac{K_{\text{av}}-EA_{\text{react}}}{K_{\text{av}}-EA_{\text{inh}}}).100$$

### Acetylcholinesterase assay

The activity of acetylcholinesterase was assayed using Ellman's method (1959) with some minor modifications. Briefly, samples containing inhibited or reactivated acetylcholinesterase (25 µL) were added to the mixture containing Britton-Robinson buffer (pH 7.4, 3 mL), acetylthiocholine iodide (7.5×10^–2^ M, 25 µL) and DTNB (1×10^–2^ M, 50 µL). The produced thiocholine reacts with DTNB to form a yellow product whose absorbance was measured at 405 nm using BA-88A biochemical semi-automatic analyzer (Shenzhen Mindray Bio-Medical Electronics). The same solution containing water instead of AChE sample served as a reagent blank.

## Results

### Complexation of obidoxime with Pd(II) ions

**Figure 2:**
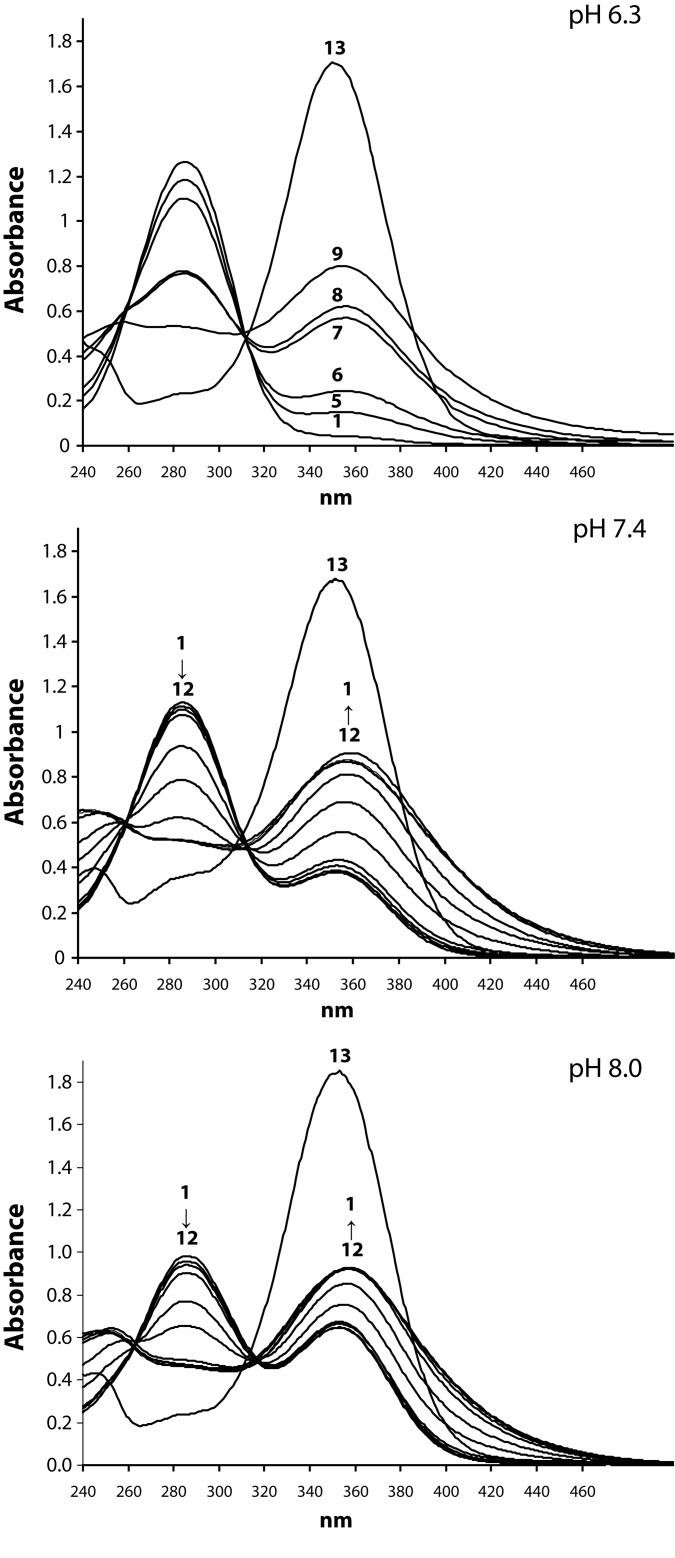
Spectral changes in the system Pd(II)-obidoxime at different pH (0 h) (obidoxime, **1**; Pd(II)-H_2_L^2+^=1:10, **2**; Pd(II)-H_2_L^2+^=1:8, **3**; Pd(II)-H_2_L^2+^=1:6, **4**; Pd(II)-H_2_L^2+^=1:4, **5**; Pd(II)-H_2_L^2+^=1:2, **6**; Pd(II)-H_2_L^2+^=1:1, **7**; Pd(II)-H_2_L^2+^=2:1, **8**; Pd(II)-H_2_L^2+^=4:1, **9**; Pd(II)-H_2_L^2+^= 6:1, **10**; Pd(II)-H_2_L^2+^=8:1, **11**; Pd(II)-H_2_L^2+^=10:1, **12**; H_2_L^2+^+Na_2_CO_3_, **13**).

The spectral changes in the Pd(II)-obidoxime system depending on pH (immediately after mixing the reagents) are presented in Figure 2. As seen, the oxime spectrum shows absorption band at 284 nm, while Pd(II)-containing species, due to formation of oximate anions (π-π* transitions within aromatic system – Gillam *et al.*, 1970; Odzak *et al.*, 2007), give rise of absorbance at 358 nm. The oximate ions obtained in the presence of Na_2_CO_3_ absorbed at 354 nm.

**Figure 3:**
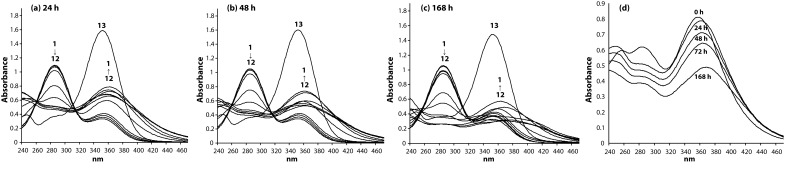
Changes in the system Pd(II)-obidoxime at pH 7.4 at the 24^th^ h (a), 48^th^ h (b) and 168^th^ h (c). Time-depending spectra of Pd(II)-obidoxime = 2:1, pH 7.4 (d) (obidoxime, **1**; Pd(II)-H_2_L^2+^ = 1:10, **2**; Pd(II)-H_2_L^2+^ = 1:8, **3**; Pd(II)-H_2_L^2+^ = 1:6, **4**; Pd(II)-H_2_L^2+^ = 1:4, **5**; Pd(II)-H_2_L^2+^ = 1:2, **6**; Pd(II)-H_2_L^2+^ = 1:1, **7**; Pd(II)-H_2_L^2+^ = 2:1, **8**; Pd(II)-H_2_L^2+^ = 4:1, **9**; Pd(II)-H_2_L^2+^ = 6:1, **10**; Pd(II)-H_2_L^2+^ = 8:1, **11**; Pd(II)-H_2_L^2+^ = 10:1, **12**; H_2_L^2+^ + Na_2_CO_3_, **13**).

The spectral changes at pH 7.4 were monitored spectrophotometrically up to 1 week after mixing the reagents. Representative spectra are given in Figure 3.

The conditional stability constant (*β’*) of complex species formed at different pH (0 h) and at pH 7.4 (24^th^ h) can be calculated (Eq. 3) based on the following complexation reaction:

*n* Pd^2+^ + H_2_L^2+^ → complex

3 $$\beta '=\frac{C_{\text{complex}}}{C_{Pd^{2+}}^{n}.C_{H_{2}L^{2+}}}$$

The main problem in the system investigated, as seen from Figures, is that the equilibrium is never shifted to the pure complex. For this reason its individual absorption spectrum is experimentally unknown and the quantitative analysis by means of UV-Vis spectroscopy is impossible by using classical methods for data processing. One of the possibilities is to apply the FiNAl procedure, which is based on resolution of overlapping bands technique, specially developed for quantitative analysis of such systems (Antonov & Nedeltcheva, 1996; Antonov & Petrov, 2002). On applying this approach, we were able to obtain the molar parts of unreacted (*x*_H_2_L^2+^_) and complexed (*x*_*complex*_) oxime species in each solution taking into account the initial pH-dependent conversion of obidoxime to oximate anions (*x*_L_). As seen from the equations below (Eqs. 4–5), molar fractions can be easily derived from the total concentration of the free ligand:

4 $$C_{H_{2}L^{2+}}^{0}=C_{H_{2}L^{2+}}+C^{*};C^{*}=C_{L}+C_{\text{complex}}$$

5 $$1=x_{H_{2}L^{2+}}+x^{*};x^{*}=x_{L}+x_{\text{complex}}$$

where (*C* ^0^_H_2_L^2+^_) is the total concentration of obidoxime, while *C*_H_2_L^2+^_, *C*_L_ and *C*_*complex*_ are the equilibrium concentrations of the free unreacted ligand, the initially formed oximate, and the complex, respectively, at given experimental conditions. The molar part of the complex was calculated from the total molar part of oximate anions (x*) (Eq. 6).

6 $$x_{\text{complex}}=x^{*}-x_{L}$$

Following this transformation, the stability constant can be presented as follows (Eq. 7–8):

7 $$\beta '=\frac{x_{\text{complex}}}{x_{H_{2}L^{2+}}}.\frac{1}{C_{Pd^{2+}}^{n}}$$

and then under logarithmic conditions

8 $$\mathrm{lg}\beta '=\mathrm{lg}\frac{x_{\text{complex}}}{x_{H_{2}L^{2+}}}-n\mathrm{lg}C_{Pd^{2+}}$$

where the number of coordinated Pd(II) ions (*n*) and the stability constant (*β’*) can be easily calculated from the plot lg $\mathrm{lg}\frac{x_{\text{complex}}}{x_{H_{2}L^{2+}}}$ *vs.* lg. $\mathrm{lg}C_{Pd^{2+}}$.

**Figure 4:**
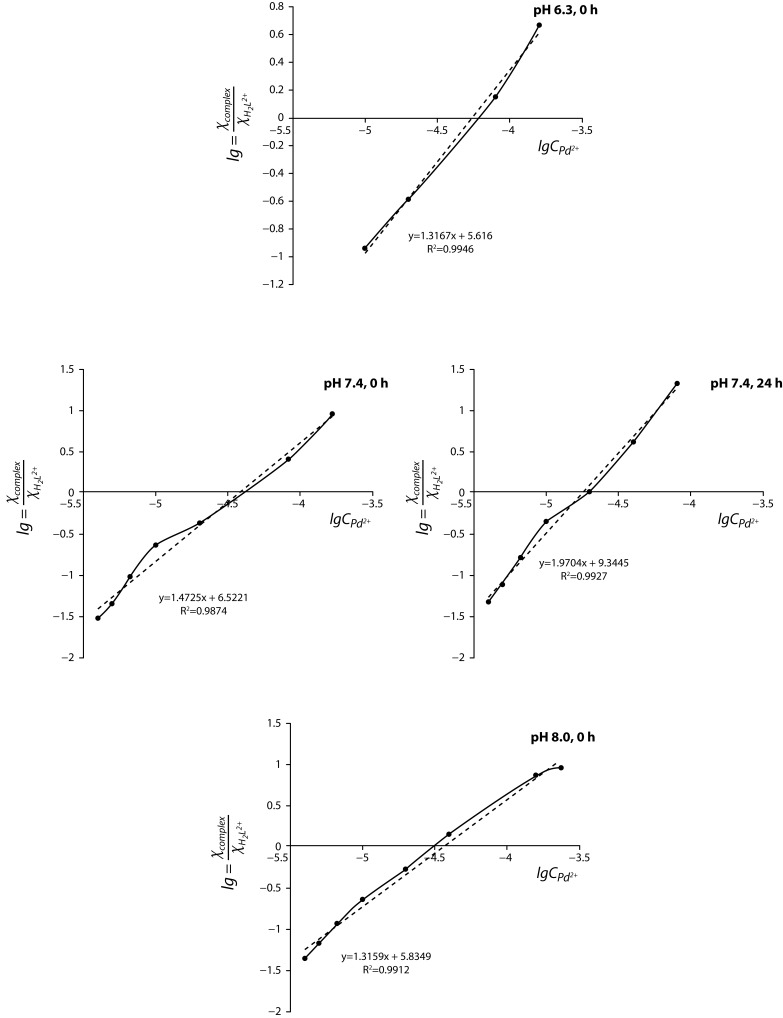
Graphical plot lg (*x*_*complex*_)/(*x*_*H*2*L*_^2+^) *vs.* lg *C*_*Pd*_^2+^ (the conversion of initial obidoxime to oximate depending on pH was taken into account according to Eqs. 4–6).

The data were treated using the above listed protocol and the results obtained immediately after preparing the reaction mixtures (pH 6.3, 7.4, 8.0) and 24 hours later (pH 7.4) are presented in Figure 4. The results of the linear fitting are summarized in Table 1.

**Table 1:** Number of palladium(II) ions bound to obidoxime (*n*) and conditional stability constant (β‘) of Pd(II)-obidoxime at different pH

pH, time	*n*	*β’*
6.3, 0 h	1.3	4.1×10^5^
7.4, 0 h	1.5	3.3×10^6^
7.4, 24 h	2.0	2.2×10^9^
8.0, 0 h	1.3	6.8×10^5^

### Reactivation of inhibited acetylcholinesterase

Paraoxon applied at 2×10^–8^ M was found to decrease the rat brain acetylcholinesterase activity with 61%±3% as compared to controls. The same inhibitor concentration was used to study the effect of obidoxime and its palladium(II)-containing species to evaluate their reactivation ability. The results showed that obidoxime (at 4×10^–5^ M) afforded 64%±5% (0 h) and 60%±6% (24 h) reactivation of AChE, while Pd(II) complex species were much less active, with a reactivation ability of 16%±2% (0 h) and 0% (24 h), respectively. The inorganic palladate(II) did not show any reactivation properties against paraoxon-inhibited enzyme.

## Discussion

Obidoxime (LuH-6, Toxogonin) discovered by Lüttringhaus and Hagedorn (1964) is a chemical antidote against nerve agent poisoning (tabun, sarin, VX) in civilians, as well as against some insecticides including paraoxon (Inns & Leadbeater, 1983; Kim *et al.*, 2006; Petroianu *et al.*, 2007b; Kuca *et al.*, 2009). Paraoxon is an organophosphorus compound with high human toxicity and its acute effects include nausea, diarrhea, excessive salivation, pupillary constriction, bronchoconstriction, muscle twitching, convulsions, coma, respiratory failure (Gosselin *et al.*, 1984).

In general, the main disadvantages of AChE reactivators as therapeutics are their specificity, along with their fast elimination and low bioavailability in the organism. In order to improve the effect of AChR, we decided to modify obidoxime into the form of coordination compound, aiming to ensure slower decomposition of complex species and subsequently to increase the amount of the active compound in the organism. It is known that oxime-containing compounds react with metal ions to form complex species, yet these reactions were used mainly for quantitative determination of oximes in various formulations (Karljiković-Rajić *et al.*, 1987; Karljiković-Rajić *et al.*, 1988; Karljiković-Rajić *et al.*, 1990; Korićanak *et al.*, 1990; Karljiković-Rajić & Rajkovic, 1997). Moreover, to the best of our knowledge, systems containing metal ions and obidoxime have not been studied so far as to their potential ability to restore the activity of inhibited acetylcholinesterase.

First we prepared a series of Pd(II)-obidoxime solutions containing different amounts of Pd(II) ions (Pd(II)-H_2_L^2+^=1:10–10:1) and recorded their UV-Vis spectra immediately after mixing the reagents. The spectral data (Figure 2) revealed that, independently on pH, a deprotonation of the ligand occurred in the presence of Pd(II) ions. The absorbance of solutions at Pd(II)-H_2_L^2+^≥2:1 does not change significantly at higher excess of metal cations. Upon addition of Pd(II) ions, obidoxime (λ_max_=284 nm) gradually converts to corresponding oximate species (λ_max_=354 nm) but the spectral changes differ from those observed in the presence of inorganic base Na_2_CO_3_. The data obtained suggest that the deprotonated obidoxime was engaged in the coordination with palladium(II) ions.

At pH 7.4, the spectral changes were monitored up to one week after mixing the reagents (Figure 3). The results showed that equilibrium reaction was completed within 24 hours, followed by disruption of the formed complex species, especially at high excess of Pd(II) ions. The time-dependence of complexation reaction can be clearly demonstrated by the example (Pd(II)-obidoxime=2:1) shown in Figure 3d.

**Figure 5:**
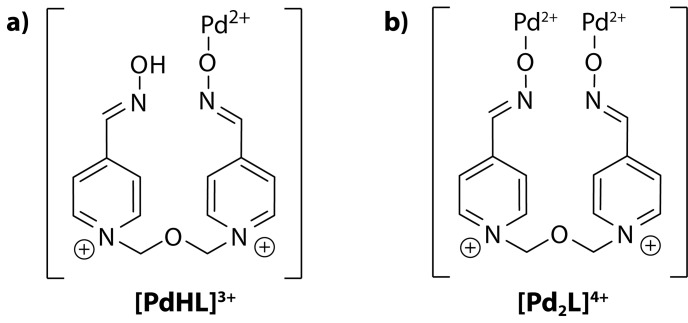
Proposed structures of Pd(II)-obidoxime complexes in solution.

The calculation of the number of Pd(II) ions (*n*) and of the conditional stability constant (*β‘*) (Table 1) revealed that the complex species of composition [PdHL]^3+^ predominantly existed immediately after mixing the reagents (*n*=1), confirmed also by the saturation method (data not shown). At the given experimental conditions, the calculated values of *β‘* are in agreement with data reported by other researchers (Karljiković-Rajić *et al.*, 1987; Karljiković-Rajić & Rajkovic, 1997). From the results obtained, the authors suggest the formation of mononuclear complex species where obidoxime is acting in a monodentate coordination mode through its oximate moiety (Figure 5a). Subsequent studies of the Pd(II)-obidoxime system at pH 7.4 showed that within 24 hours a second Pd(II) ion coordinated forming the final product of the composition [Pd_2_L]^4+^ (*n*=2). Based on the chemical structure of the ligand and the calculated metal-to-ligand molar ratio of 2:1, one can suppose that obidoxime functions as a monodentate bridging ligand forming binuclear complex species (Figure 5b). The stability of [Pd_2_L]^4+^increases significantly reaching the value of lg*β‘* 9.34. Due to the high solubility of obidoxime and its complex species we have so far not been able to isolate new compounds in solid state for their precise structure characterization. Further studies in this respect are in progress.

The formation of new obidoxime species raised the question for their potential to reactivate inhibited rat brain acetylcholinesterase. As mentioned above, the quantitative formation of [PdHL]^3+^ (0 h) and [Pd_2_L]^4+^ (24 h) species at pH 7.4 reaches their maxima at metal-to-ligand molar ratio Pd(II)-obidoxime = 2:1 and does not change significantly at higher excess of metal cations. For that reason we selected this reaction mixture to evaluate reactivation properties of modified obidoxime. The data showed that neither of the complex species did reactivate significantly the paraoxon-inhibited rat brain acetylcholinesterase at *in vitro* conditions, as compared to the non-coordinated ligand. The lack of reactivation ability could be explained by the considerable stability of complexes in solution as well as by the deprotonation of oxime groups essential for the disinhibition of the enzyme (Holstege *et al.*, 1997; Carlton *et al.*, 1998; da Silva Gonçalves *et al.*, 2011).

The *in vitro* acetylcholinesterase assay revealed that complexation of obidoxime with Pd(II) ions cannot be applied as efficient reactivation system in the case of paraoxon poisonings. Still the question about *in vivo* efficacy of this system remains open.

## Conclusion

The complexation of the acetylcholinesterase reactivator obidoxime with Pd(II) cations at pH 7.4 leads to formation of [PdHL]^3+^ and [Pd_2_L]^4+^ complex species, depending on the reaction time. The experimental data showed that new species possessed very low *in vitro* reactivation ability against paraoxon-inhibited rat brain acetylcholinesterase as compared to the non-coordinated obidoxime.
